# CDK9 INHIBITORS: a promising combination partner in the treatment of hematological malignancies

**DOI:** 10.18632/oncotarget.28473

**Published:** 2023-08-07

**Authors:** Daniel Morillo, Gala Vega, Victor Moreno

**Affiliations:** ^1^Division of Hematology, START Madrid-FJD, Hospital Fundación Jiménez Díaz, Madrid, Spain; ^2^Division of Oncology, START Madrid-FJD, Hospital Fundación Jiménez Díaz, Madrid, Spain

**Keywords:** cyclin-dependent kinases (CDK), CDK9, hematological malignancies

## Abstract

Most hematological malignancies are characterized by overexpression of certain cancer promoting genes, such as MYC, MCL1 and cyclin D1. Preclinical studies in animal models have shown that CDK9 inhibitors supress the transcription of these anti-apoptotic and pro-survival proteins, and suggest their potential synergism with other drugs. In its first in-human trial, enitociclib demonstrated clinical activity in a small cohort of patients with high grade B lymphoma with MYC and BCL2 and/or BCL6 rearrangements, inducing complete responses in 2 of 7 subjects (29%) in monotherapy. These data suggest CDK9 inhibitors could play a role in the treatment of hematological diseases and could be a great ally when combined with other therapeutic approaches.

## INTRODUCTION

Cyclin-dependent kinases (CDK) belong to a family of serine/threonine kinases that need to form heterodimeric complexes with cyclins to perform their functions. These kinases are involved in multiple processes within cells, including cell cycle, apoptosis, transcription and differentiation. These kinases are often overexpressed in different malignancies, making them potential targets for new drugs [1]. Among transcription-associated CDKs, CDK9, along with cyclin T, forms the main component of positive transcription elongation factor b (PTEFb). This complex phosphorylates RNA polymerase II (RNAPII) to stimulate transcription elongation of most protein coding genes [2, 3]. Inhibition of P-TEFb plays a key role in tumors that continuously produce short-lived proteins such as MYC, myeloid cell leukemia 1 protein (MCL1) and cyclin D1, which are well known to be involved in the development of most hematological malignancies [4]. The observed clinical activity of non-selective CDK inhibitors was attributed primarily to their CDK9 activity [5]. The first generation of pan-CDK inhibitors targeting CDK9, which included flavopiridol and dinaciclib, showed suboptimal efficacy and their development was therefore suspended [6, 7]. A second generation of inhibitors were further developed, and exhibited more specific affinity for CDK9. The first in this class of CDK9 inhibitors, atuveciclib, was evaluated in two clinical studies, involving patients with advanced solid tumors and in subjects with relapsed acute myeloid leukemia (AML) [8, 9]. Both studies demonstrated high incidences of neutropenia that could not be managed with granulocyte colony-stimulating factor across different dose schedules. This led to dose-limiting toxicity at sub-therapeutic doses and resulted in discontinuation of the drug. However, further optimization of atuveciclib led to the development of a highly potent and more selective CDK9 inhibitor, enitociclib (VIP152, formerly BAY 1251152) [10]. In human xenograft tumor models of AML, enitociclib showed promising *in vivo* antitumor efficacy as a single agent, with a favorable tolerability profile across various dosing schedules, including weekly intravenous dosing [10].

In the first in-human trial (NCT02635672), carried out by Diamond and collaborators [11], enitociclib showed evidence of clinical efficacy with a low toxicity profile in advanced malignancies. Remarkably, a subgroup of patients treated for lymphoma with MYC and BCL6 and/or BCL2 rearrangements, so-called high-grade B-cell lymphoma (HGBL), also showed clinical benefit [12]. The study enrolled 37 patients with solid tumors or lymphomas refractory to all available therapies. They received enitociclib monotherapy as a 30-minute intravenous infusion, given once-weekly, every 21 days. The escalating doses were 5, 10, 15, 22.5 and 30 mg until disease progression or unacceptable toxicity. The maximum tolerated dose of enitociclib was defined as 30 mg. The most common treatment-related adverse events were grade 1/2 in severity. Grade 3/4 events were infrequent and independent of dose, except for neutropenia (21.6%), which was satisfactorily managed with granulocyte colony-stimulating factor, G-CSF, but required dose reduction in 6 cases. The pharmacokinetic and pharmacodynamic profiles were consistent with the *in vivo* results. Comparison to baseline revealed downregulation of MYC, MCL1 and PCNA mRNA levels at all doses tested. This downregulation was dose- and time-dependent, with maximal pathway inhibition achieved at the two highest doses (22.5 and 30 mg).

With respect to its antitumor efficacy, enitociclib showed no objective activity in a cohort of solid tumors (not selected based on MYC expression) for 7 patients achieved stable disease (23%) 2 of those responses were durable over time (9.5 months in a patient with pancreatic cancer and 16.8 months in a patient with salivary gland cancer). The most remarkable responses were observed in the HGBL cohort. Of those 7 patients, 2 (29%) showed durable complete metabolic responses that lasted more than 2 and 3 years, respectively.

**Figure 1:**
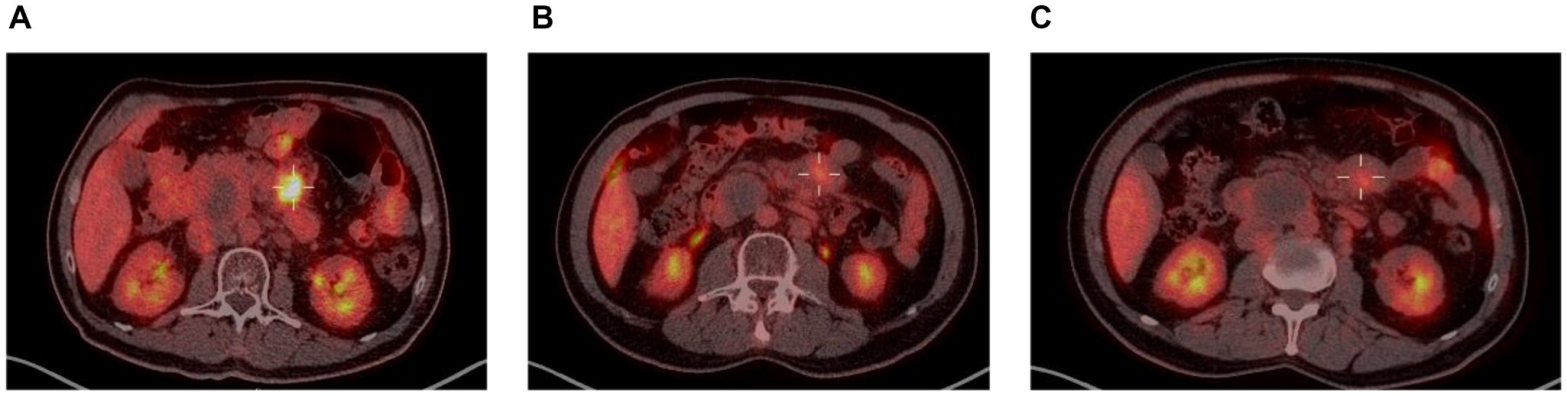
PET scan at screening (**A**), 5 months (**B**) and 8 months on treatment (**C**).

**Figure 2:**
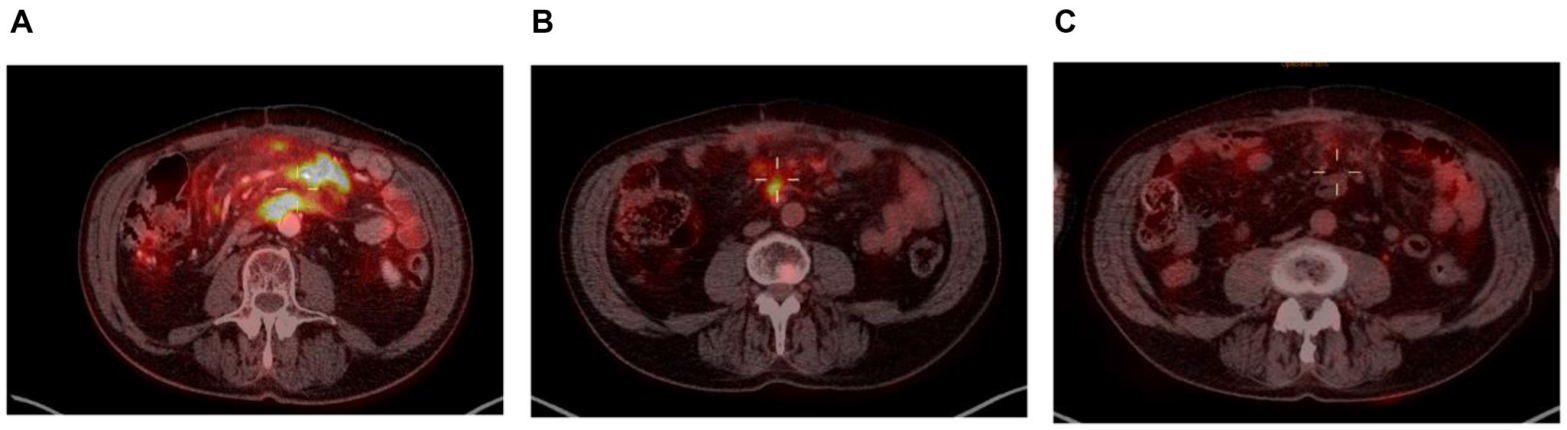
PET scan at screening (**A**), at 6 months (**B**) and at 7 months (**C**).

Taking a closer look at those 2 patients, both had localized disease at inclusion (Ann-Arbor stage II), and their responses to enitociclib progressively deepened over time. Partial responses were achieved after 5 and 6 months and complete metabolic responses (CMR) at 7 and 8 months from the first dose received (Figures 1 and 2). These patients stopped treatment (as a result of the COVID-19 pandemic during which follow-up became more difficult) after 20 and 36 months in complete remission, respectively. And after 6.4 and 4.8 years, their CMRs continue to be sustained as of today. These findings are indicative of the antitumor activity of this new CDK9 inhibitor against HGBL. However, it requires time to achieve this effect, likely because the drug’s mechanism of action requires time to fully inhibit the production of short-lived proteins and thus induce clinical responses.

HGBLs are aggressive diseases with a high proliferation index and, unfortunately, most patients progress within the first weeks of treatment. Consequently, monotherapy with a CDK9 inhibitor would seems to be insufficient to overcome these highly proliferative tumors. For that reason, combined treatment strategies may be a reasonable approach to improve efficacy. Indeed, the rapid development of new drugs to treat DLBCL/HGBL in recent years offers a broad spectrum of treatment possibilities. For instance, the approval of CAR-T therapy has changed the present treatment paradigm in the relapse/refractory (R/R) setting, obtaining outstanding response rates. However, there are two main issues to be considered. First, long manufacturing processes lead to a higher risk of disease progression in patients awaiting therapy. Second, only about 40% of patients achieve durable remission [13]. Bispecific monoclonal antibodies are a great off-the-shelf alternative in patients with R/R DLBCL/HGBL, inducing complete responses in approximately 40% of patients, even after CAR-T [14], though long-term follow-up will be required to confirm whether the response rate is maintained over time. Several monoclonal antibodies and antibody drug conjugates, including tafasitamab [15] and loncastuximab tesirine [16], both targeting CD19, and polatuzumab, which targets antiCD79b, have been approved [17]. Zilovertamab vedotin is a new antibody-drug conjugate that targets ROR1 antigen and shows promising preliminary results [18]. Among the targeted small molecule therapies, the efficacy of cereblon E3 ligase modulators (CELMoDs) has been confirmed when used in monotherapy and in combination [15]. BTK inhibitors are being tested for ABC DLBCL [19] and MALT, and CARD11 inhibitors are under development. The new generation CDK9 inhibitors, especially enitociclib, could be a good combination partner with these drugs due to its low toxicity profile and different mechanism of action, which could result in synergistic effects. For instance, the second-generation CDK2/9 inhibitor fadraciclib (CYC065) was shown to reduce MCL-1 protein levels and to induce apoptosis in chronic lymphocytic leukemia cells. Moreover, preclinical work suggests fadraciclib overcomes microenvironment-mediated protection and exerts synergism between CDK2/CDK9 inhibitor and venetoclax [20].

## CONCLUSION

In summary, most hematological malignancies are characterized by overexpression of certain cancer promoting genes, such as MYC and MCL1. CDK9 inhibitors are relatively new drugs that inhibit transcription of these anti-apoptotic and pro-survival proteins. Different preclinical studies have confirmed the activity of CDK9 inhibitors in animal models and their potential synergism with other drugs. In its first in-human trial, enitociclib demonstrated clinical activity in a small cohort of patients with HGBL harboring MYC and BCL2 and/or BCL6 rearrangements, and complete responses were achieved in 2 of 7 subjects (29%) in monotherapy. These data suggest CDK9 inhibitors could play a role in future treatments of hematological diseases and could be a great ally when combined with other therapeutic approaches.
